# Rhythmic lipid and gene expression responses to chilling in panicoid grasses

**DOI:** 10.1093/jxb/erae247

**Published:** 2024-05-29

**Authors:** Sunil K Kenchanmane Raju, Yang Zhang, Samira Mahboub, Daniel W Ngu, Yumou Qiu, Frank G Harmon, James C Schnable, Rebecca L Roston

**Affiliations:** Center for Plant Science Innovation, University of Nebraska-Lincoln, Lincoln, NE, USA; Center for Plant Science Innovation, University of Nebraska-Lincoln, Lincoln, NE, USA; Center for Plant Science Innovation, University of Nebraska-Lincoln, Lincoln, NE, USA; Department of Biochemistry, University of Nebraska-Lincoln, Lincoln, NE, USA; Center for Plant Science Innovation, University of Nebraska-Lincoln, Lincoln, NE, USA; Department of Statistics, Iowa State University, Ames, IA, USA; Plant Gene Expression Center, USDA-ARS, Albany, CA, USA; Department of Plant and Microbial Biology, University of California, Berkeley, CA, USA; Center for Plant Science Innovation, University of Nebraska-Lincoln, Lincoln, NE, USA; Department of Agronomy and Horticulture, University of Nebraska-Lincoln, Lincoln, NE, USA; Center for Plant Science Innovation, University of Nebraska-Lincoln, Lincoln, NE, USA; Department of Biochemistry, University of Nebraska-Lincoln, Lincoln, NE, USA; RIKEN Center for Sustainable Resource Science, Japan

**Keywords:** Chilling stress, diel rhythms, lipid abundance, lipid unsaturation, panicoid grasses

## Abstract

Chilling stress threatens plant growth and development, particularly affecting membrane fluidity and cellular integrity. Understanding plant membrane responses to chilling stress is important for unraveling the molecular mechanisms of stress tolerance. Whereas core transcriptional responses to chilling stress and stress tolerance are conserved across species, the associated changes in membrane lipids appear to be less conserved, as which lipids are affected by chilling stress varies by species. Here, we investigated changes in gene expression and membrane lipids in response to chilling stress during one 24 h cycle in chilling-tolerant foxtail millet (*Setaria italica*), and chilling-sensitive sorghum (*Sorghum bicolor*) and *Urochloa* (browntop signal grass, *Urochloa fusca*, lipids only), leveraging their evolutionary relatedness and differing levels of chilling stress tolerance. We show that most chilling-induced lipid changes are conserved across the three species, while we observed distinct, time-specific responses in chilling-tolerant foxtail millet, indicating the presence of a finely orchestrated adaptive mechanism. We detected rhythmicity in lipid responses to chilling stress in the three grasses, which were also present in *Arabidopsis thaliana*, suggesting the conservation of rhythmic patterns across species and highlighting the importance of accounting for time of day. When integrating lipid datasets with gene expression profiles, we identified potential candidate genes that showed corresponding transcriptional changes in response to chilling stress, providing insights into the differences in regulatory mechanisms between chilling-sensitive sorghum and chilling-tolerant foxtail millet.

## Teaser

Chilling stress triggers distinct, time-specific lipid and transcriptional responses in cold-tolerant foxtail millet compared with their non-tolerant relatives, elucidating species-specific adaptations, and conserved circadian rhythms across plants.

## Introduction

Climate change has increased the frequency and severity of extreme weather events, threatening future food supply (Ray *et al.*, 2013). Major crop species important for global food security, such as maize (*Zea mays*), sorghum (*Sorghum bicolor*), and rice (*Oryza sativa*), are sensitive to chilling stress owing to their tropical origin, limiting their geographical distribution and productivity in temperate climates (Taylor and Rowley, 1971; Lyons, 1973). Chilling stress is a major stress experienced by plants during their life cycle, hindering energy metabolism and growth, most notably by reducing the activity of enzymes associated with photosynthesis and the energy-demanding production of protective proteins and substances (Hurry *et al.*, 2002; Kaplan and Guy, 2004). Moreover, plants must endure daily and seasonal temperature fluctuations and unexpected extreme variations (Larran *et al.*, 2023). Plants have devised various strategies to cope with such environmental challenges.

At the cellular level, low-temperature stress leads to increased membrane rigidity and impaired containment of cytosolic contents, resulting in cell death (Uemura *et al.*, 1995; Zoldan *et al.*, 2012). Changes in glycerolipids, major components of cell membranes, include membrane lipid polyunsaturation (Hugly and Somerville, 1992; Miquel *et al.*, 1993), changing the ratio of lipid head groups, and removing membrane-destabilizing lipids in response to low temperature (Moellering *et al.*, 2010; Barnes *et al.*, 2016). The contribution of unsaturated fatty acids to membrane fluidity at different temperatures and their role in protecting the photosynthetic machinery from photoinhibition under chilling stress are well known (Nishida and Murata, 1996). However, no consistent changes in membrane lipid abundance during chilling stress have been reported across species. These discrepancies in changes in lipid compositions or content during stress may be due to differences in the duration and/or intensity of the applied stress, time-of-day effects, and/or genetic and physiological differences across species (Kenchanmane Raju *et al.*, 2018).

Plant responses to these stressful environments can vary greatly at the transcriptional level, although a core set of transcriptional responses is mostly conserved across species (Kenchanmane Raju *et al.*, 2018). Notably, most studies of cold tolerance in the *Pooideae* grass subfamily of the *Poaceae* [including wheat (*Triticum aestivum*), barley (*Hordeum vulgare*), and rye (*Secale cereale*)] have revealed chilling adaptive mechanisms that are not shared by closely allied subfamilies within the grasses, such as the *Ehrhartoideae* (which includes rice). This lack of conservation suggests that different plant lineages have adapted to growth in temperate environments using distinct genetic and physiological mechanisms. Panicoid grasses, comprising many important crops such as maize, sugarcane (*Saccharum officinarum*), switchgrass (*Panicum virgatum*), sorghum, and foxtail millet (*Setaria italica*), exhibit a range of sensitivities to cold temperatures (Hope and McElroy, 1990; Dohleman and Long, 2009; Kenchanmane Raju *et al.*, 2018). The repeated acquisition and loss of chilling tolerance within this subfamily (Sandve *et al.*, 2008; Pardo and VanBuren, 2021) make it an ideal system to study the conserved and species-specific adaptation strategies for chilling tolerance.

Sorghum, an important crop in the arid and semi-arid regions of the world, originated in the semi-arid tropics of Africa and quickly spread into other parts of the world, including India, China, and the USA (Doggett and Majisu, 1968). Due to its tropical origin, sorghum is susceptible to chilling (Burow *et al.*, 2011). While landraces and wild relatives are important gene pools for adaptive traits such as biotic stress resistance and abiotic stress tolerance (Brozynska *et al.*, 2016), the limited availability of standing genetic variation and newer cropping environments require the transfer of stress adaptation mechanisms from closely related stress-adapted species. Like sorghum, foxtail millet is also a grain crop domesticated from a panicoid grass. However, foxtail millet was initially domesticated in northeast China from a wild grass, green foxtail (*Setaria viridis*) that grows in temperate climates where low-temperature stress is more common (Bennetzen *et al.*, 2012; G. Zhang *et al.*, 2012; Yang *et al.*, 2012).

Orthologous genes, even within closely related taxa, can show differential regulation of chilling stress-responsive gene expression between maize and sorghum, or between maize, sorghum, and eastern gamagrass (*Tripsacum dactyloides*) (Y. Zhang *et al.*, 2017; Yan *et al.*, 2019), suggesting that orthology alone is not a reliable predictor of stress-induced gene expression in related species (Meng *et al.*, 2021). It can therefore be challenging to narrow down target genes for chilling stress tolerance in sorghum and related chilling-sensitive species.

To overcome these challenges, we designed a time-course experiment to account for potential time-of-day variation and tested the relationship between chilling stress tolerance, changes in membrane glycerolipid contents, and evolutionary relatedness using three panicoid grasses. Browntop signal grass (*Urochloa fusca*, Urochloa hereafter) is a grass closely related to foxtail millet that is less chilling tolerant. Urochloa and sorghum are more distantly related and have similar susceptibility to chilling stress. In this study, we profiled the changes in membrane lipid contents and composition and in transcript levels in these three species using paired time-course measurements of control and chilling stress conditions. We identified differentially regulated genes increased in chilling-tolerant foxtail millet, including *3-KETOACYL-COA SYNTHASE 1* (*KCS1*), known for its effects on chilling stress tolerance. We also showed that correlating lipid abundance changes with gene expression profiles allowed the identification of lipid metabolic genes responding to chilling within a species, such as sorghum’s *DIGALACTOSYL DIACYLGLYCEROL DEFICIENT 1* (*SbDGD1*). These genes have potential application in engineering chilling tolerance in sorghum and related chilling-sensitive grasses.

## Materials and methods

### Plant growth and chilling treatment

Seeds for the reference genotypes for sorghum (*Sorghum bicolor*, *BTx623*), maize (*Zea mays*, *B73*), Urochloa (*Urochloa fusca*, *LBJWC-52*), and foxtail millet (*Setaria italica*, *Yugu1*) were grown in a Percival growth chamber (E-41L2) with 60% relative humidity, with a 12 h light/12 h dark photoperiod and a target temperature of 29 °C during the day and 23 °C at night. Chilling stress was applied to 12-day-old seedlings, when collars of two leaves are visible. Immediately at the end of the light period, seedlings were moved to a second growth chamber with identical photopheriod settings and a target temperature of 6 °C. Each sample represents a pool of above-ground tissue from at least three seedlings. Samples were harvested from the control conditions and chilling stress-treated plants at the designated time points after the onset of chilling stress. *Arabidopsis thaliana* (Arabidopsis) of the Columbia ecotype were planted as described (Barnes *et al.*, 2023), and grown under a 16 h light/8 h dark photoperiod to enhance the effect of initial chilling in this frost-tolerant species. Chilling stress was applied to 4-week-old rosettes, at the transition to flowering. Immediately at the end of the light period, seedlings were moved to a second growth chamber with equivalent identical settings and a target temperature of 6 °C

For lipid analysis, whole shoot tissue of seedlings was removed at the soil level, excluding the coleoptile of grasses. Samples were harvested at 0 min (immediately before reducing the chamber temperature to 6 °C), 10 min, 3 h, 6 h, 12 h, and 24 h. The 10 min sample was taken 10 min after the chamber air temperature reached 6 °C, ~20 min past time 0. Whole shoot tissue excluding the coleoptile was collected at the soil level. Due to a combination of sample loss during processing and outlier analysis, the number of represented biological replicates changed by the lipid species analyzed (always ≥3 biological replicates for each growth/treatment trial). The tissue was quickly and gently submerged in 1 ml of ice-cold extraction solvent (2:1:0.1 v/v/v methanol:chloroform:formic acid) in a 2 ml tube and shaken on a bead beater at 4000 inversions min^–1^ in 30 s intervals with intervening ice incubations until the tissue was thoroughly disrupted. Lipid extraction continued following a modified Bligh and Dyer protocol (Mahboub *et al.*, 2021). Following extraction, lipids were concentrated and stored at –80 °C under nitrogen. Lipids were separated as described in Wang and Benning (2011) with the following modifications. A 10% lipid spot was loaded in the corner of each TLC plate that did not see solvent, which served as a control for total fatty acids and was used to make internal comparisons.

A two-dimensional TLC plate was used for separation. In the first dimension, a mixture of chloroform:methanol:ammonium hydroxide, (130:50:10, v/v/v) was used as solvent and, in the second dimension, chloroform:methanol:acetic acid: water (85:12.5:12.5:4, v/v/v/v) was used as a solvent. A separate one-dimensional thin-layer chromatogram was used to separate non-polar triacylglcyerol, with petroleum ether:diethyl ether:acetic acid (80:20:1, v/v/v) as solvent. Lipids were identified by retention time compared with standards purchased from Avanti Polar Lipids. The remaining analysis was precisely done as described in Barnes *et al.* (2016).

The statistical analysis of lipid data involved several steps. Outliers were assessed at two levels. Firstly, for fatty acid abundance, a robust regression of outlier removal (ROUT) analysis was performed at a 10% threshold using GraphPad v9.5.0 to eliminate any misidentified peaks or anomalies. Any outliers detected at this step were removed from further analysis. Second, the relative mole percentages of each lipid were calculated and normalized to the total fatty acids present. The resulting mole percentages were then screened for outliers using one interquartile distance from the median for each lipid class for each genotype at each temperature. Asterisks denote statistical significance (*P*≤0.05), determined by fitting a mixed model, with Tukey’s correction for multiple tests. Due to a combination of manual error causing sample loss during processing and outlier analysis, the exact number of represented biological replicates changed by the lipid species analyzed, and was always between three and eight biological replicates for each split among at least two separate growth trials.

The double bond index (DBI) was calculated using the formula: (X:1)×1+(X:2)×2+(X:3)×3/100, where X represents the relative mole % of 16:*n* and 18:*n* fatty acids, and *n* denotes one, two, or three double bonds. Multiple comparisons were adjusted using Tukey’s multiple comparisons test when comparing between genotypes.

### Measurement of CO_2_ assimilation rates

Seedlings were grown and stress treated as above, with the modification that small plastic caps were placed over sorghum, foxtail millet, and Urochloa seedlings to prevent them from becoming too tall to fit into the LI-COR measurement chamber. After 0, 1, or 8 d of chilling treatment, seedlings were allowed to recover in the greenhouse overnight under control conditions, and CO_2_ assimilation rates were measured the next morning using the LI-6400 portable photosystem unit under the following conditions: photosynthetically active radiation (PAR) 200 µmol mol^−1^, CO_2_ at 400 µmol mol^−1^, with a flow rate of 400 µmol mol^−1^, and humidity at greenhouse conditions. Whole seedlings readings were measured for sorghum, foxtail millet, and Urochloa after covering the pots with clay and using the LI-COR’s Arabidopsis chamber. Readings for maize were measured using the leaf clamp attachment which was always placed on the second leaf at a position 3 cm above the ligule. Leaf area was measured using the LI-3100C area meter.

### RNA isolion and RNA-seq analysis

Total RNA was isolated from paired samples collected at 0.5, 1, 3, 6, 16, and 24 h after the onset of chilling. Library construction was performed following the protocol described by Y. Zhang *et al.* (2017). Sequencing was conducted at the Illumina Sequencing Genomics Resources Core Facility at Weill Cornell Medical College. Raw sequencing data are available through the NCBI (http://www.ncbi.nlm.nih.gov/bioproject) under accession number SRA: SRP090583 and BioProject: PRJNA344653. Summary statistics for all the libraries are provided in Supplementary Table S1. Adapters were removed from the raw sequence reads using cutadapt v1.6. RNA-seq reads were mapped to genome assemblies downloaded from Phytozome (v13): v3.1 (sorghum) and v2.2 (foxtail millet). RNA-seq reads from each species were aligned using GSNAP (Wu *et al.*, 2016), and fragments per kilobase of transcript per million mapped reads (FPKM) values were obtained using cufflinks v2.2.1 (Trapnell *et al.*, 2010).

### Syntenic orthologs in sorghum and foxtail millet

A final set of 9778 syntenic orthologous gene pairs between sorghum and foxtail millet was calculated from the previously published list of syntenic orthologs (Zhang *et al.*, 2017) after filtering for SD <0.4 and *r*^2^ >0.1 of the FPKM values. Clustering was performed using ‘correlation’ from R packages ‘fpc’ (https://cran.r-project.org/web/packages/fpc/fpc.pdf; https://cran.r-project.org/web/packages/cluster/cluster.pdf). To observe treatment effects, the ratio between treatment FPKM and control FPKM was determined for the time-course. A permutation test was performed by keeping the sorghum gene constant and randomly assigning a different foxtail millet gene 100 times to determine the appropriate *r*^2^, SD, and number of clusters. Syntenic orthologs found within the same clusters were considered co-expressed orthologs (CEOs), while syntenic orthologs found in different clusters were considered as differentially expressed orthologs (DEOs).

### Identification of differentially regulated orthologs

The FPKM values were measured from three biological replicates each for sorghum and foxtail millet under control and cold treatment at six time points (0.5, 1, 3, 6, 16, and 24 h). Similar to the cluster analysis, the treatment over control (T/C) FPKM ratios at each time point for sorghum and foxtail millet were calculated and treated as a response. A linear mixed model (LMM) was used to model the T/C ratios as a relationship between the species identity and sampling time under chilling stress on the same set of syntenic orthologous gene pairs used in the cluster analysis. Let yijkl denote the T/C ratio of the ith gene from the kth species and the lth biological replication at the jth time point, where j=1–6 to represent the six time points, k=1, or 2 to represent the two species: sorghum and foxtail millet, and l=1, 2, or 3 to represent the three biological replicates. There were a total of six biological replicates in the study, three from sorghum and three from foxtail millet. We modeled the biological replication as a random effect. For the ith gene, conditioned on this random replication effect, the response yijkl is normally distributed with mean µijkl and variance σ 2 i. The expected T/C ratio µijkl was linearly related to the species, time, and their interactions as µijkl=νi + αij + βik + γijk + ηikl for ηikl ∼N(0, θ2 i), (1) where νi is the intercept; αij and βik stand for the main effect of time and species for the ith gene, respectively; γijk is the interaction between time and species, denoting different patterns of expression between the two species over time; and ηikl is the random effect for the biological replicates, which is assumed to be normally distributed with mean 0 and variance θ 2 i. Note that the interaction effect γijk in the model (1) stands for the difference of the T/C ratios over time between sorghum and foxtail millet. The non-zero interaction effect represents different patterns of T/C ratios changing over time between the two species, while the zero γijk indicates a similar trend of the responses of the two species. Those genes with non-zero interaction effect are defined as differentially regulated orthologs (DROs) and those with zero interaction effect are called comparably regulated orthologs (CROs). In order to identify the DROs, we considered the hypotheses Hi,0: γijk=0 for all j, k versus Hi,a: γijk 6=0 for some j, k (2) for each gene. Estimation of γijk and its associated standard error were obtained by the ‘lmer’ function in the R package lme4. Wald test statistics were conducted for hypothesis (2), and the associated *P*-value for each gene was calculated. Benjamini and Hocheberg multiple test correction was applied to control for false discovery rates (FDRs) >0.001. Those pairs with an FDR <0.001 were considered as DROs, and those with an FDR >0.01 were considered as CROs.

### Lipid genes in sorghum and foxtail millet.

A manually curated list of Arabidopsis genes known to be involved in lipid pathways was first created using the Aralip database (http://aralip.plantbiology.msu.edu/pathways/pathways). The sorghum and foxtail millet genes were then matched to the Arabidopsis lipid genes using the published best Arabidopsis hits for the sorghum and foxtail millet genome on Phytozome (v13). Each sorghum and foxtail millet hit was matched with their respective syntenic ortholog in the other species, creating a list of syntenic orthologous pairs of lipid genes in sorghum and foxtail millet.

### Gene expression and lipid heatmaps

FPKM values and lipid abundance and unsaturation were normalized by linear transformation such that the minimum value within the time-series turned into a zero and maximum values are turned to one. All other values get transformed into decimals between 0 and 1. Heatmaps were generated using the heatmap2 function in R.

### Identification of rhythmicity in lipid abundance and expression of lipid-related genes

Rhythms in lipid abundance were identified with the ‘circa_single’ method in CircaCompare (package version 0.1.1) in R (version 4.3.0) with default settings (Parsons *et al.*, 2020). Differences in lipid abundance waveforms were detected with the ‘circacompare’ method in the same package. FPKM values representing expression at 3 h intervals over 72 h for the 356 lipid metabolism-associated genes that were syntenic between sorghum and foxtail millet were derived from previously published transcriptomes of comparably staged third-leaf stage seedlings from sorghum, foxtail millet, and maize (Lai *et al.*, 2020). Genes in the 356 metabolism-associated dataset exhibiting differential rhythmicity between temperature treatments (i.e. cold treatment versus no treatment) or genotypes (sorghum versus foxtail millet) were identified with the R package LimoRhyde (Singer and Hughey, 2019) in Bioconductor (Gentleman *et al.*, 2004). LimoRhyde reports Benjamini and Hochberg *q*-values (Benjamini and Hochberg, 1995) of the rhythmicity of a gene and differential rhythmicity for genes shared between the two datasets. Statistical significance for either rhythmicity or differential rhythmicity was set at a *q*-value of ≤0.05. Foxtail millet genes were keyed to their sorghum synteologs for LimoRhyde identification of differential rhythmicity between sorghum and foxtail millet genes.

## Results

### Foxtail millet is chilling tolerant compared with other panicoid grasses

**Fig. 1.**
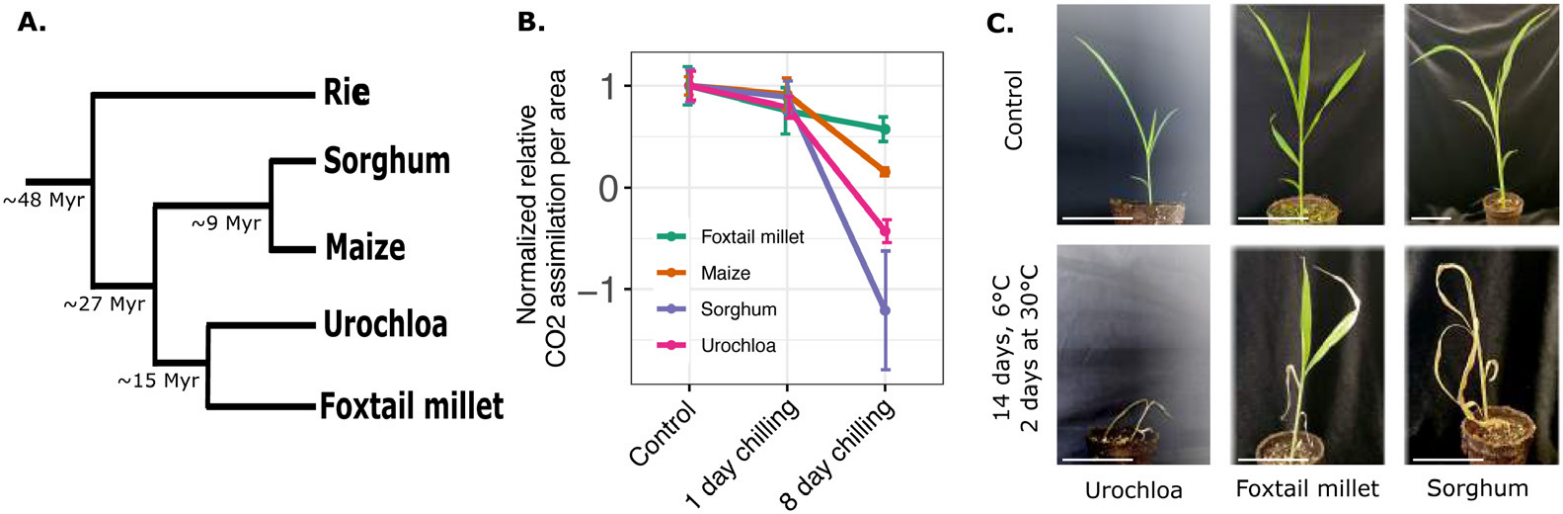
Foxtail millet is a chilling-tolerant representative of the panicoid grass clade. (A) Evolutionary relationships of the four species evaluated with rice as an outgroup. Numbers indicate divergence time as reported in Zhang *et al.* (2017) and Pessoa-Filho *et al.* (2017). (B) Normalized relative CO_2_ assimilation rates for panicoid grass species with differing degrees of sensitivity or tolerance to chilling stress. CO_2_ assimilation was measured after treatment at 6 °C for the indicated times below (1 d or 8 d) followed by an overnight return to 30 °C of ~10 h. Leaf area was measured immediately after assimilation. Individual data points are jittered on the *x*-axis to avoid overlap. Lines indicate mean values for each species across three replicates, and whiskers represent standard error of the mean. (C) Phenotypic response of foxtail millet, Urochloa, and sorghum to 6 °C chilling stress for 14 d, followed by 2 d of return to 30 °C. Scale bars, 6 cm.

Chilling stress causes structural transitions in biological membranes of cold-susceptible plants. These membrane changes cause respiration abnormalities and photosynthetic CO_2_ and O_2_ exchange rates (Lyons, 1973; Larcher, 1995). Lower photosynthesis for prolonged periods, continuing for hours or days, is an essential identifier of chilling susceptibility (Larcher, 1995). Here, we used CO_2_ assimilation rates to quantitatively assess differences in chilling tolerance among closely related panicoid grasses (Fig. 1A; Y. Zhang *et al.*, 2017). Accordingly, we measured 12-day-old seedlings grown under control conditions (29 °C during the day and 23 °C at night) and after exposure to chilling treatment (6 °C) in growth chambers for 1 d or 8 d. After 8 d of chilling stress, sorghum, Urochloa, and maize showed lower values for CO_2_ assimilation compared with the control time point, indicating impaired photosynthetic activity. In fact, sorghum and Urochloa seedlings had dead leaves, which was reflected in the negative CO_2_ assimilation values (Fig. 1B). Foxtail millet showed moderate impairment in its photosynthetic rate as its CO_2_ assimilation measurements remained at ~55% of control levels even after 8 d of stress, indicating higher tolerance to chilling (Fig. 1B) consistent with its native range and center of domestication in Northern China (H.Y. Lu *et al.*, 2009; G. Zhang *et al.*, 2012). Based on these photosynthetic measurements, we classified the four panicoid species into two categories: chilling-susceptible—sorghum, Urochloa, and maize; and chilling-tolerant—foxtail millet. Prolonged stress clearly differentiated tolerance levels in foxtail millet. Following 2 weeks of chilling stress at 6 °C and 2 d of return to normal growing temperatures, Urochloa and sorghum seedlings were dead while foxtail millet seedlings looked healthier with fewer necrotic leaves (Fig. 1C).

### Foxtail millet membranes have distinct responses to chilling stress

**Fig. 2.**
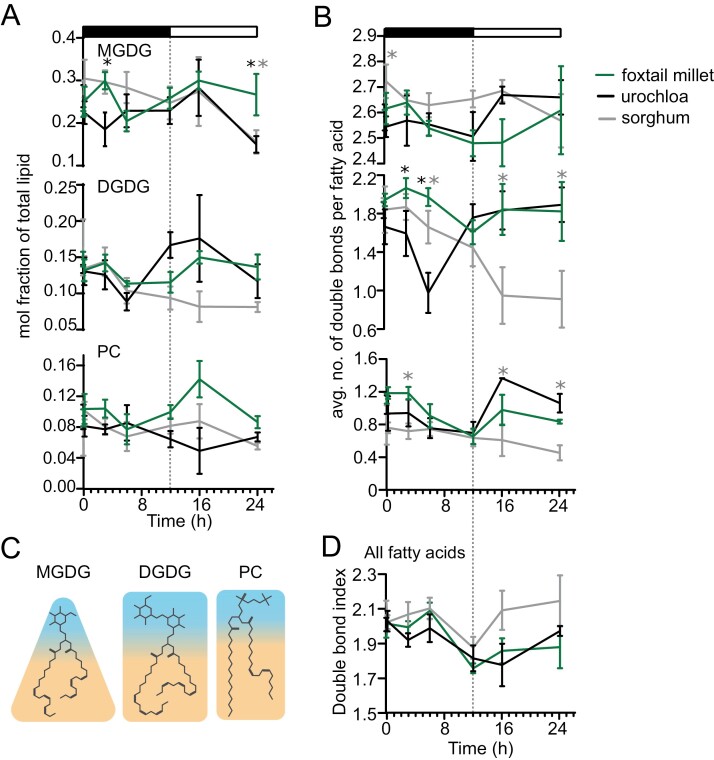
Lipid responses to chilling include effects related to genetic distance and chilling tolerance. The relative abundance of specific lipid species exhibits multiple sequential changes in the first 24 h of exposure to chilling stress. In all panels, the *x*-axis indicates the time in hours (h) (A) Mole percent abundance of lipids relative to all fatty acid-containing lipids for the following lipid classes: monogalactosyldiacylglycerol (MGDG), digalactosyldiacylglcyerol (DGDG), and phosphatidylcholine (PC) in foxtail millet, Urochloa, and sorghum. (B) Unsaturation index, calculated as the average number of double bonds per fatty acid for all fatty acid-containing lipids: MGDG, DGDG, and PC. *P*-values were determined using Fisher’s least significant difference (LSD). *P-value <0.05, **P-value <0.01, and ***P-value <0.001. (C) Structural models of major lipids MGDG, DGDG, and PC, where blue indicates the hydrophilic head group and orange indicates the hydrophobic tail group. (D) Total unsaturation index, calculated as the average number of double bonds per fatty acid for all fatty acid-containing lipids. For all samples, *n* was between three and eight biological replicates.

Many cellular membrane systems are damaged in response to chilling (Lyons, 1973; Nishida and Murata, 1996), and changes in membrane lipid compositions are required to achieve chilling tolerance (Uemura *et al.*, 1995; Zoldan *et al.*, 2012). We profiled membrane lipids from sorghum, Urochloa, and foxtail millet seedlings grown under control and chilling stress conditions. We hypothesized that patterns unique to foxtail millet and not present in both sorghum and Urochloa potentially stem from the difference in chilling tolerance among the species. Likewise, patterns in foxtail millet that are shared by Urochloa but not sorghum are likely to reflect their closer evolutionary relationship. We collected samples for lipid profiling at 10 min, 3 h, 6 h, 12 h, 16 h, and 24 h following the onset of chilling stress. Of the 11 lipids measured (Supplementary Table S1), nine lipids exhibited 24 h rhythmic accumulation (rhythmic hereafter) in at least one species (Supplementary Table S2) (Singer and Hughey, 2019). In foxtail millet, all three major membrane lipids, monogalactosyldiacylglycerol (MGDG, LimoRhyde *q*-value=0.07), digalactosyldiacylglycerol (DGDG, LimoRhyde *q*-value=0.04), and phosphatidylcholine (PC, LimoRhyde *q*-value=0.03) were found to be rhythmic (Fig. 2; Supplementary Table S2). PC was rhythmic in all three species, while triacylglyceride (TAG) and phosphatidylglycerol (PG) were rhythmic in sorghum and Urochloa. Major lipids such as DGDG and PC were rhythmic in foxtail millet and Urochloa (Fig. 2A), suggesting a strong influence of genetic relatedness on major lipid abundance patterns. However, a foxtail millet-specific increase in MGDG abundance was observed at 24 h post-chilling stress compared with sorghum and Urochloa (*P*-value=0.003 and *P*-value=0.004, respectively) (Fig. 2A). Further, we tested the difference in rhythmicity in PC and DGDG between species using CircaCompare analysis (Parsons *et al.*, 2020). The time at which the metabolites (response variable) reach peak abundance (phase) is significantly different for PC in all three species (Supplementary Table S3). Mesor, a rhythm-adjusted mean, is significantly different in foxtail millet compared with sorghum (*P*-value=0.001) and Urochloa (*P*-value=0.006). We also examined the variation in rhythmicity of lipid saturation levels among species. In foxtail millet, DGDG, MGDG, PC, and total saturation exhibited significant rhythmicity, whereas in Urochloa only PC and total saturation showed significant rhythmicity. In sorghum, only total lipid saturation displayed significant rhythmicity (Supplementary Table S3). These results show that rhythmic lipids across species differ in their rhythmicity or peak and mesor values, suggesting species-specific control of rhythmicity in lipid content and composition.

Similar headgroup and fatty acid tail sizes in lipids such as PC and DGDG favor strong interactions that stiffen membranes during cold stress, while smaller headgroups such as those of MGDG promote fluidity at lower temperatures (Fig. 2C). We initially expected a dip in PC and DGDG levels alongside corresponding climbs in PE and MGDG during chilling, but observed no such trend within the first 24 h for any species (Fig. 2A; Supplementary Fig. S1). This prompted us to shift our focus to fatty acid unsaturation, as it affects the head-to-tail size ratio and influences membrane fluidity. Further, low temperature-induced increases in fatty acid polyunsaturation of membrane lipids are associated with greater membrane fluidity and increased chilling tolerance (Quinn *et al.*, 1989; Miquel *et al.*, 1993). We detected significant differences in DGDG unsaturation levels in foxtail millet compared with Urochloa following 3 h of chilling stress and relative to Urochloa and sorghum at 6 h of chilling stress, indicating a foxtail millet-specific early stress response (Fig. 2B; Supplementary Table S4). We observed similar species-specific differences in lipid unsaturation levels for minor lipids such as phosphatidylethanolamine (PE), phosphatidylinositol (PI), PG, phosphatidylserine (PS), and sulfoquinovosyldiacylglycerol (SQDG) (Supplementary Fig. S1). The total lipid unsaturation index remained high for sorghum throughout the time-course, while foxtail millet and Urochloa were characterized by lower unsaturation near the end of the time-course (Fig. 2D; Supplementary Table S4). Thus, neither the bulk changes in lipid head groups nor unsaturation in these species can explain the increased low-temperature tolerance of foxtail millet in the first 24 h of chilling.

### Transcriptional changes in lipid metabolism genes are associated with lipid abundance change

In previous work, we have shown that lipid pathway genes were differentially regulated in temperate-adapted *Tripsacum dactyloides* compared with maize and sorghum in response to chilling stress and were enriched among genes experiencing rapid rates of protein sequence evolution in *T. dactyloides* (Yan *et al.*, 2019). To examine whether transcriptional changes in lipid metabolism genes match the observed patterns of lipid changes between chilling-tolerant foxtail millet and chilling-sensitive sorghum, we collected samples from sorghum and foxtail millet for transcriptome sequencing (RNA-seq) at 0.5, 1, 3, 6, 16, and 24 h after the onset of chilling stress, as well as from paired control samples not exposed to chilling stress, collected at the same time points. We employed a conventional correlation co-expression clustering method and an LMM-based method to understand the differences and commonalities in how sorghum and foxtail millet respond to chilling stress at the transcriptional level (see the Materials and methods).

We used a set of 16 796 syntenic orthologous gene pairs conserved between sorghum and foxtail millet (Zhang *et al.*, 2017). Of these, 9778 gene pairs passed an expression data quality filter of SD <0.4 and *r*^2^ >0.1 (Supplementary Table S5; see the Materials and methods). Of this filtered set, 2233 gene pairs (Supplementary Table S6) exhibited a significant species×treatment interaction effect (multiple testing-corrected FDR <0.001; Benjamini and Hochberg, 1995), indicating differences in the chilling stress-induced transcriptional response of orthologous genes between the two species. In parallel, we applied conventional correlation clustering analysis to identify co-expressed syntenic orthologous gene pairs in sorghum and foxtail millet. We used the ratio of expression values between treatment and control conditions for clustering analysis. Using a permutation test, we defined 16 clusters (see the Materials and methods; (Supplementary Fig. S2; Supplementary Table S7) and identified 2245 syntenic orthologous genes in different clusters as being CEOs. We classified the remaining 7533 syntenic orthologs as non-co-expressed orthologs and referred to them as correlation cluster-differentially regulated orthologs (CC-DROs; Supplementary Table S8). Clusters 2, 4, 6, and 14 had more sorghum genes, while clusters 1, 3, 5, 7, 8, 9, 10, 11, and 13 had a higher proportions of foxtail millet genes. Clusters 12, 15, and 16 had a similar number of genes from sorghum and foxtail millet (Supplementary Table S7). Clusters 1, 3, 6, and 7 contained genes up-regulated at 6 h into stress, indicating a possible role in early chilling stress response. We illustrate the divergence in transcriptional responses to chilling between syntenic gene pairs in a Circos plot, in which lines that cross over between groupings in the center of the chart represent genes that are syntenic orthologs and have distinct patterns of gene expression between foxtail millet and sorghum (Supplementary Fig. S2).

We then identified high-confidence differentially regulated orthologs (HC-DROs) by taking the overlap of CC-DROs identified by the clustering method and the DROs identified with LMM (Supplementary Table S9). We determined that 1708 syntenic orthologous gene pairs overlap in the two sets, which we further used for gene ontology (GO) term enrichment analysis. GO analysis of these 1708 HC-DRO pairs revealed enrichment for two GO categories: ‘stress response’ and ‘macromolecule metabolic process’. In validation of our focus on lipids, we observed an enrichment for the GO metabolic process category, ‘lipid metabolic process’ (GO:0006629, *P*-value=0.003, Supplementary Table S10).

**Fig. 3.**
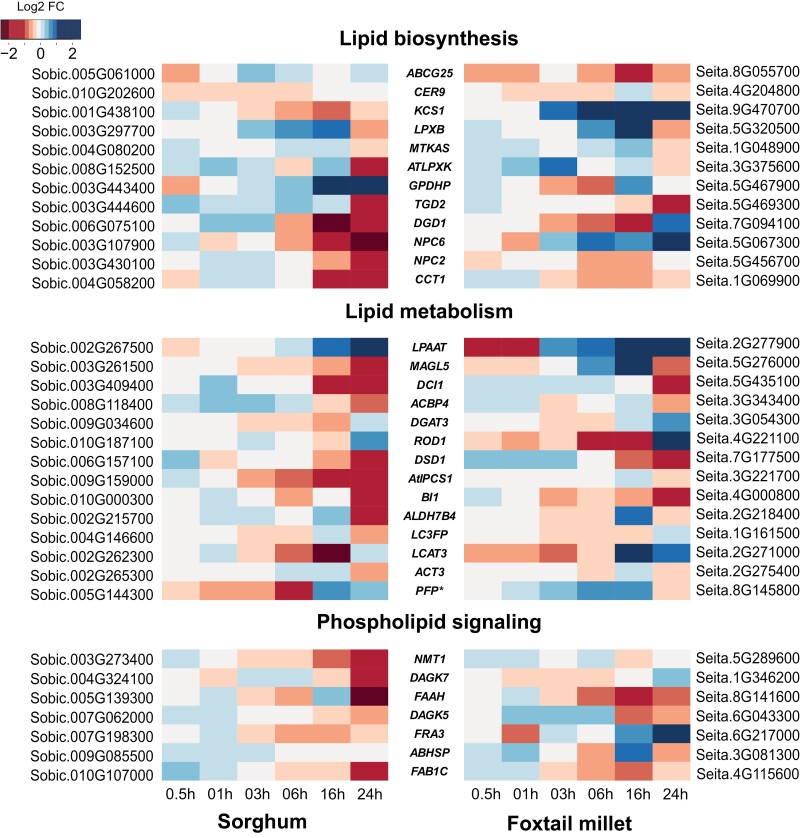
Syntenic orthologs in sorghum and foxtail millet show differential regulation during chilling stress. Heatmap representation of log2 fold change values for chilling-stressed samples compared with control in sorghum and foxtail millet at different time points. Lipid-related gene pairs that overlapped with high-confidence differentially regulated orthologs were considered and classified into lipid biosynthesis, lipid metabolism, and phospholipid signaling. Genes names in the center are suggestive only and derived from best-hit Arabidopsis genes.

We then defined a set of *a priori* candidates from the most likely set of Arabidopsis lipid genes corresponding to fatty acid and glycerolipid metabolism from the AraLipid database (http://aralip.plantbiology.msu.edu/pathways/pathways), and a corresponding set of 356 sorghum–foxtail millet gene pairs homologous to these Arabidopsis genes with syntenic orthologs in both sorghum and foxtail millet (Supplementary Table S11). The overall gene expression patterns of these 356 gene pairs revealed that lipid-related genes are mostly up-regulated under chilling treatment in chilling-tolerant foxtail millet, but not in sorghum. Of the 356 lipid-related gene pairs, 34 showed differential responses to chilling stress between sorghum and foxtail millet, with pronounced up-regulation of lipid-related gene expression in foxtail millet exposed to chilling stress (Fig. 3; Supplementary Table S12). One example of such a DRO in sorghum and foxtail millet is provided by *KCS1*, encoding an enzyme in the fatty acid elongation pathway for wax biosynthesis and involved in chilling tolerance in Arabidopsis (Chen *et al.*, 2020). The sorghum ortholog of *KCS1*, Sobic.001G438100, was down-regulated throughout the chilling stress time-course. However, the *KCS1* ortholog in the chilling-tolerant foxtail millet, Seita.9G470700, was up-regulated at later time points (Fig. 3), suggesting that the differential regulation of *KCS1* ortholog expression between sorghum and foxtail millet may be leading to the differences in chilling tolerance between the two species.

### Gene expression correlation with lipid buildup and breakdown

**Fig. 4.**
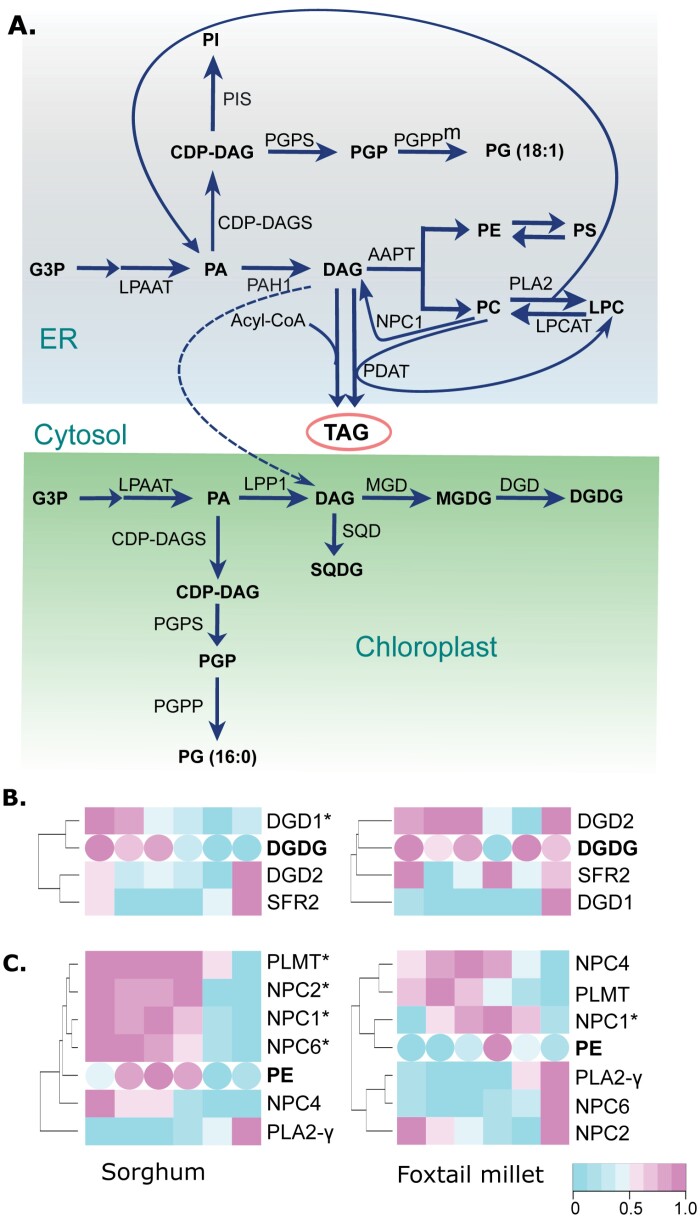
Correlation of lipid and transcript abundances in the glycerolipid biosynthesis pathway allows identification of candidate genes. (A) Diagram of the glycerolipid biosynthesis pathway, with lipid species shown in bold font and the enzymes responsible for each step denoted next to the corresponding arrows. (B, C) Heatmaps showing transcript abundance for candidate genes in lipid metabolism and abundance of lipid species (in bold) at different time points for DGDG (B) and phosphatidylethanolamine (PE) (C). Significant correlations, measured by Pearson correlation, between lipid changes and transcript abundance in either sorghum or foxtail millet are indicated by ‘*’ after the gene name.

We asked whether changes in the expression of genes in lipid pathways in foxtail millet and sorghum were consistent with changes in lipid abundance and saturation. To this end, we combined time-course lipid and gene expression profiles to understand how differential gene expression in these two species affects lipid abundance and saturation under chilling stress, using only shared time points between the two datasets. A diagram of the glycerolipid biosynthesis pathway is shown in Fig. 4A. Looking at the sorghum ortholog of Arabidopsis *DGD1*, Sobic.006G075100, its expression profile had a positive correlation with DGDG accumulation during chilling [Pearsons’s correlation coefficient (PCC) *r*=0.85, *P*-value=0.03]. However, the expression of the *DGD1* ortholog in foxtail millet was not correlated with DGDG accumulation (Fig. 4B). Similarly, the expression of *NON-SPECIFIC PHOSPHOLIPASE C1* (*NPC1*) orthologs in sorghum and foxtail millet was positively correlated with PE accumulation during chilling (PCC *r*=0.79, *P*-value=0.06; PCC *r* =0.84, *P*-value=0.03, respectively). However, the expression of *NPC2*, *NPC6*, and *PHOSPHOLIPID N METHYLTRANSFERASE* (*PLMT*) was also positively correlated with PE accumulation in sorghum but not in foxtail millet (Fig. 4C). Notably, we detected correlations between gene expression and lipid contents for lipids with species-specific changes in lipid abundance, such as MGDG and DGDG (Figs 2, 4B).

The accumulation of TAGs in plants arises from multiple sources (Du and Benning, 2016; Xu and Shanklin, 2016), and TAGs are important for low-temperature tolerance (Arisz *et al.*, 2018; J. Lu *et al.*, 2020; Klinska-Bachor *et al.*, 2023). Correlating gene expression patterns with TAG abundance was expected to shed light on the potential source of TAG during the chilling response. The expression levels of the foxtail millet ortholog to Arabidopsis *LIPID PHOSPHATE PHOSPHATASE 2* (*LPP2*), Seita.4G217800, showed a significant and positive correlation with lipid abundance in TAG accumulation during chilling stress response in foxtail millet (PCC *r*=0.86, *P*-value=0.003). By contrast, the expression levels of the sorghum ortholog to LPP2, Sobic.010G190300, showed no significant correlation with TAG accumulation (Supplementary Table S13). This finding suggests that, at least in foxtail millet, phospholipids are the primary source of chilling-stress-induced TAG accumulation. A list of specific orthologs in foxtail millet and sorghum whose expression levels were significantly correlated with the buildup and breakdown of each lipid species is provided (Supplementary Tables S13, S14).

### Conservation of chilling-induced changes in lipid composition and rhythmicity in Arabidopsis

**Fig. 5.**
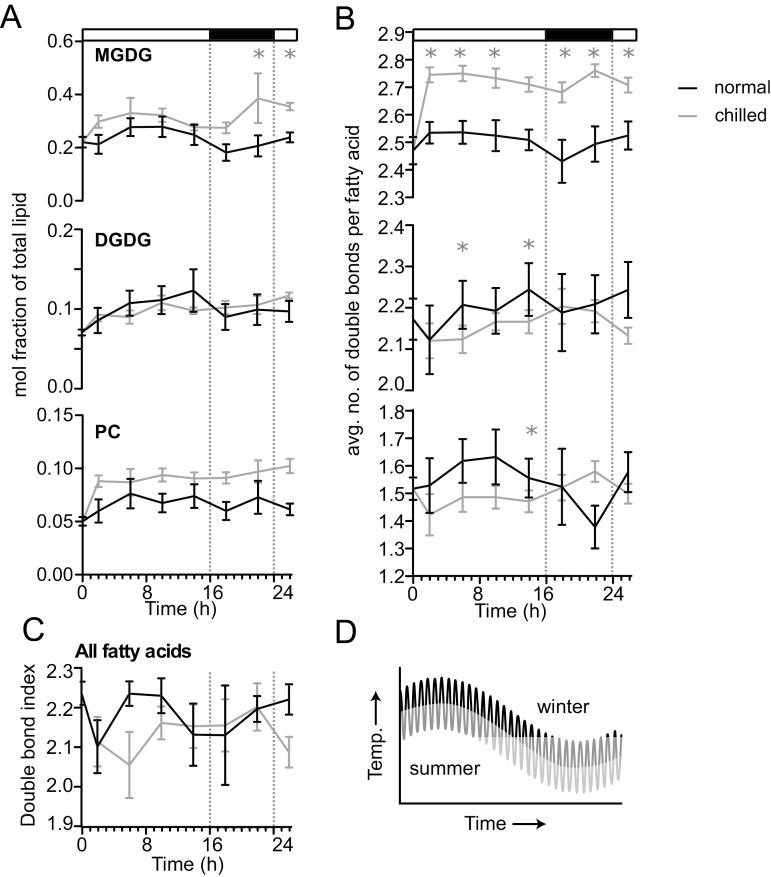
Time-series profiles of Arabidopsis lipid abundance and unsaturation during chilling or control conditions. (A) Mole percent abundance of lipids relative to all fatty acid-containing lipids for the following lipid classes: monogalactosyldiacylglycerol (MGDG), digalactosyldiacylglcyerol (DGDG), and phosphatidylcholine (PC) in Arabidopiss during chilling or control conditions. (B) Unsaturation index, calculated as the average number of double bonds per fatty acid for MGDG, DGDG, and PC. (C) Unsaturation index, calculated as the average number of double bonds per fatty acid for all fatty acid-containing lipids. Significant *P*-values determined using Fisher’s least significant differences. *P-value <0.05, **P-value <0.01, and ***P-value <0.001. (D) Diagram showing daily and seasonal fluctuation of temperature during the plants’ growth cycle. For all samples, *n* was between three and eight biological replicates.

Previous reports of lipid diel rhythmicity, or rhythmicity on a 24 h cycle, described changes of specific lipids under normal growing conditions in Arabidopsis (Maatta *et al.*, 2012; Nakamura *et al.*, 2014). To test if differences in rhythmicity were observable between normal and chilling conditions, we quantified representative lipids from Arabidopsis seedlings across a time-course with paired control samples and chilling stress samples collected immediately before chilling stress (0 h), and after 2, 6, 10, 14, 18, 22, and 26 h of stress. DGDG levels remained constant during normal conditions or chilling stress, whereas MGDG levels were slightly higher upon chilling stress compared with control conditions, reaching statistical significance at 22 h and 26 h into stress (Fig. 5A). This increase in MGDG levels at the late time points was similar to the significant increase in MGDG after 24 h of exposure to stress in chilling-tolerant foxtail millet compared with chilling-susceptible sorghum and Urochloa (Fig. 2). PC levels increased and remained higher across the entire time course (Fig. 5A; Supplementary Table S15). However, PC saturation under chilling stress conditions was only distinguishable from control samples at a few time points (Fig. 5B, C). We detected significant rhythmicity in MGDG levels in control conditions (Supplementary Table S16, rhythmic *P*-value=0.005) using the ‘circa_single’ method in CircaCompare analysis (Parsons *et al.*, 2020), and the pattern differed by the end of the 24 h sufficiently to decrease the rhythmicity prediction below significance. Similar rhythmicity changes were observed for DGDG and PC saturation levels (Fig. 5B). CircaCompare analysis supported the significance of DGDG saturation rhythmicity during chilling (Supplementary Table S16, rhythmic *P*-value=0.017), but not under control conditions, and PC saturation rhythmicity during control (Supplementary Table S16, rhythmic *P*-value=0.013), but not during chilling conditions. Interestingly, the amplitudes of major lipids—MGDG, DGDG, and PC—were much lower in Arabidopsis compared with the three grasses. These results suggest the conservation of chilling tolerance-induced changes in lipid content and composition and rhythmic patterns of lipids across grasses and Arabidopsis despite 150 million years of divergence between monocots and eudicots (Brendel *et al.*, 2002).

### Lipid-related genes exhibit expression rhythmicity

**Fig. 6.**
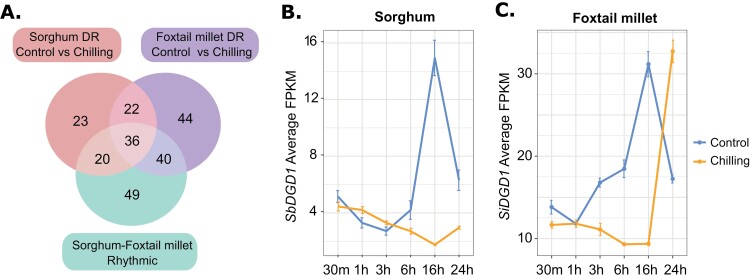
Comparison of rhythmicity and differential rhythmicity in sorghum and foxtail millet lipid-related genes. (A) Venn diagram showing the extent of overlap between rhythmic genes in sorghum and/or foxtail millet identified by LimoRhyde analysis under control conditions (244 genes in the green circle), sorghum differentially rhythmic (DR) genes under control conditions compared with chilling treatment (101 genes in the pink circle), and foxtail millet DR genes under control conditions compared with chilling treatment (142 genes in the purple circle). The union of these three datasets is 36 genes representing rhythmic genes under control conditions that change their rhythmicity in response to chilling temperature in both foxtail millet and sorghum. (B, C) An example of a rhythmic gene *DGD1* showing a similar diel expression pattern under control conditions and changed rhythmicity during chilling stress response in sorghum (B) and foxtail millet (C). *DGD1* expression is shown as average FPKM values from three biological replicates and the standard error of the mean.

The conservation of chilling-induced lipid changes implied that chilling tolerance may have evolved in response to daily rhythms in temperature, and then adapted to seasonal variation (Fig. 5D). To determine which sorghum and foxtail millet lipid-related genes exhibit 24 h rhythms, we examined the patterns of 356 sorghum–foxtail millet lipid-related gene pairs in a previously published 72 h RNA-seq time-course (Lai *et al.*, 2020). We identified 224 sorghum and 189 foxtail millet genes in this set as being rhythmically expressed. Of these, 145 pairs were rhythmic in both sorghum and foxtail millet. We then used the LimoRhyde package (Singer and Hughey, 2019) to identify those genes with rhythmic expression under control conditions in our datasets. This analysis indicated that 131 sorghum and 204 foxtail millet lipid-related genes had rhythmic expression patterns under control conditions (Supplementary Table S17). Further, we employed LimoRhyde to test for differences in rhythmic expression, or differential rhythmicity, for each gene between the control and chilling treatments in sorghum and foxtail millet. We identified 142 foxtail millet lipid-related genes and 101 sorghum lipid-related genes displaying differential rhythmicity between the control and chilling treatments (Supplementary Table S17). Among the 58 lipid-related gene pairs that showed differential rhythmicity between control and chilling stress in both sorghum and foxtail millet, 36 showed rhythmic expression under control conditions in both species (Fig. 6A). These lipid-related gene pairs are rhythmic genes that change their rhythmicity patterns under chilling treatment and probably represent shared targets in sorghum and foxtail millet for chilling stress-induced alterations in expression.

An example of such a lipid-related gene whose rhythmic expression under control conditions is altered during chilling stress in both sorghum and foxtail millet was *DGD1* (Fig. 6B, C). Sorghum and foxtail millet *DGD1* showed similar rhythmic patterns of expression under control conditions. However, their rhythm and magnitude of expression change significantly during chilling stress in both species. Of note, *DGD1* was identified as a differentially regulated ortholog in Fig. 3. In addition, *SbDGD1* expression during chilling stress was positively correlated with DGDG abundance in sorghum. The foxtail millet *DGD1* ortholog did not show such a correlation, suggesting species-specific changes in their response to chilling stress. These results indicate that differences in the diel regulation of lipid-related genes in sorghum and foxtail millet may lead to differential responses to chilling stress.

## Discussion

Panicoid grasses represent an interesting clade with repeated gain or loss of chilling tolerance, reflecting parallel adaptation strategies in different lineages within the clade. Using representative chilling-susceptible sorghum and chilling-tolerant foxtail millet allowed us to identify changes to transcript and lipid levels that are likely to be functionally linked to variation in chilling tolerance between the related species. We included Urochloa, a chilling-sensitive panicoid grass that is more closely related to foxtail millet, as a control for the large evolutionary divergence between foxtail millet and sorghum.

Here, we assembled time-course datasets for transcript levels and lipid metabolic profiling in three panicoid grasses with different genetic relatedness and tolerance to chilling stress to understand whether and how changes in the composition of membrane lipids and corresponding changes in gene expression contribute to chilling tolerance in foxtail millet. Most changes in lipid content and composition were consistent across the three species (Fig. 2A, B, D), probably representing shared responses to chilling stress due to their genetic relatedness. Urochloa appears to behave more like foxtail millet than it does like chilling-susceptible sorghum, probably an effect of its short evolutionary distance compared with foxtail millet. By comparing sorghum with Urochloa, we were able to tease out a small subset of lipid metabolic changes that are unique to foxtail millet, the most chilling-tolerant panicoid grass tested in this study (Fig. 2). These results also indicate that lipid unsaturation is unlikely to be the source of chilling tolerance in foxtail millet, as it is similarly adjusted in all three species during the first 24 h of chilling (Fig. 2B; Supplementary Fig. S2).

There is little consensus in reports of changes in lipid content and composition in response to cold stress across land plants (Kenchanmane Raju *et al.*, 2018). These discrepancies in lipid-related changes may reflect inherent genetic and physiological differences in how individual species respond to chilling stress; alternatively, they may stem from varying experimental designs and variation due to sampling time. Evidence is fast emerging for the role of circadian clock regulation in coordinating dynamic plant responses to daily and seasonal environmental fluctuations (Espinoza *et al.*, 2010; Panter *et al.*, 2019). However, daily rhythms in lipid metabolism had not previously been reported for important clades of crops such as panicoid grasses under chilling conditions. Notably, rhythmic changes in lipid composition and gene expression during chilling stress are not similar across species, suggesting that a general strategy is not to stop or slow down the circadian clock during stress; rather, plants may have developed species-specific strategies to overcome these challenges. Our time-series dataset of changes affecting lipids during chilling stress in three grasses allowed us to uncover rhythmic patterns of lipid abundance and unsaturation. Moreover, our lipid dataset reveals chilling tolerance-related changes in lipid abundance and unsaturation at specific time points, potentially explaining part of the difficulty in extracting conserved patterns for lipids across species in previous reports involving one or a few time points. We also detected rhythmicity in the expression of lipid metabolic genes in both sorghum and foxtail millet. The rhythmic nature of changes in lipids and specific changes during chilling stress were similar between grasses and Arabidopsis, in contrast to other published studies that show differences. These findings highlight the importance of time-series datasets to account for diel cycles in uncovering conserved features of chilling stress responses across large phylogenetic distances.

The diel variation in lipid abundance observed in Arabidopsis (Fig. 5A) was roughly half as intense as that observed in the panicoid grasses (Fig. 2A). This disparity raises the question of why grasses exhibit such pronounced fluctuations in lipidome composition relative to Arabidopsis. While previous studies in maize, a related panicoid grass, highlight a rhythmic accumulation of lipids and lipid precursors (Li *et al.*, 2020), a potential explanation for the stronger amplitudes in grasses may lie in their C_4_ photosynthetic architecture. Long (1999) demonstrated higher peak photosynthetic activity in C_4_ species, and photosynthate can theoretically be directly converted into lipids (Clark and Schwender, 2022). Notably, engineered sorghum strains can produce substantial amounts of lipid (Vanhercke *et al.*, 2019). This potentially higher lipid influx in grasses could explain the more pronounced diel oscillations observed. Additionally, our inability to detect consistent rhythmicity in all major lipids of Arabidopsis may be linked to its overall lower amplitude. However, a definitive understanding of the species-specific variation in lipidome dynamics requires further comparative studies encompassing both transcriptomes and lipidomes, preferably across diverse genotypes and chilling stress conditions with many time points.

One of our most intriguing observations was the diel variation in fatty acid double bond content, observed in both panicoid grasses and Arabidopsis (Figs 2D, 5C; Supplementary Tables S3, S4, S15, S16). Manipulating desaturase activity to increase unsaturation is a proven strategy for boosting low-temperature tolerance in various plant species, including grasses and Arabidopsis (Shi *et al.*, 2018; Wang *et al.*, 2019; Wang *et al.*, 2021). Similarly, loss of desaturase activity reduces plant low-temperature tolerance (Kunst *et al.*, 1989; Hugly and Somerville, 1992; Miquel *et al.*, 1993, Chen and Thelen, 2013). However, the relationship between fatty acid unsaturation and low temperature is more nuanced than this implies, as analysis of 10 published low-temperature treatments revealed a decrease in the double bond index with dropping temperatures (Kenchanmane Raju *et al.*, 2018). In our hands, foxtail millet and Urochloa exhibited a transient rise in the total lipid double bond index within the first 10 min of chilling exposure, potentially reflecting an adaptation to initial membrane stiffening (Fig. 2D; Supplementary Fig. S4; Supplementary Tables S4, S15). However, this trend did not persist throughout the 24 h chilling period for any species tested (Figs 2D, 5C; Supplementary Tables S4, S15), suggesting that other mechanisms, such as phytosterols or membrane–protein interactions, play a role in maintaining membrane fluidity alongside unsaturation during the first 24 h of chilling.

Previous studies in Arabidopsis have documented diel variations in lipid saturation under normal growth conditions (Ekman *et al.*, 2007; Maatta *et al.*, 2012; Nakamura *et al.*, 2014), which has been attributed to light-dependent fatty acid synthesis (Browse *et al.*, 1981; Kim *et al.*, 2023). Congruently, our analysis revealed diel variation in total fatty acid saturation across all three grass species (Fig. 2B; Supplementary Tables S3, S4), suggesting a similar underlying biological mechanism. While previous research in Arabidopsis focused on highly unsaturated PC molecules peaking in the dark (Ekman *et al.*, 2007; Maatta *et al.*, 2012; Nakamura *et al.*, 2014), our study did not analyze specific PC molecules. However, we observed rhythmicity in PC saturation across foxtail millet, Urochloa, and Arabidopsis, with overall saturation levels declining in the dark (Figs 2B, 5B; Supplementary Tables S3, S4, S15, S16). In contrast, sorghum did not exhibit similar PC saturation rhythmicity, indicating species-specific differences. Furthermore, species-specific variations in saturation rhythmicity were also observed in MGDG and DGDG among the grass species (Supplementary Tables S3, S4).

Combined lipidomic and transcriptomic analysis has been used to unravel transcriptional regulation of lipid metabolism during chilling stress responses in maize (Gu *et al*., 2017). Our results show species-specific differences in transcriptional correlation with lipid metabolic changes, suggesting complex regulation of metabolic perturbations involved in plants’ response to environmental challenges. We propose that this approach with chilling-susceptible and chilling-tolerant species can enable the identification of specific genes whose transcript levels are correlated with changes in lipid metabolites, in response to chilling stress. However, our experimental design may miss non-syntenic genes that may have acquired novel chilling-induced changes in their expression. We identified species-specific differences in the extent of correlation between lipid-related gene expression and changes in lipid abundance, potentially informing the flux of fatty acids. For example, we discovered that the expression of the foxtail millet *LPP2* is tightly correlated with TAG abundance. LPP2 generates DAG from phospholipids in the endoplasmic reticulum, DAG is a precursor to TAG (Fig. 4A), implying that phospholipids are the primary source of TAG in foxtail millet during chilling response. By contrast, the expression of the sorghum ortholog of *LPP2* was not correlated with TAG abundance, suggesting species-specific differences in membrane lipid funneling to TAG between these two species during chilling stress. *NPC1* expression (Fig. 4) and PE levels are another example of an unexpected lipase influencing lipid levels. *NPC1* expression was correlated with PE accumulation in foxtail millet and sorghum. *NPC1* produces DAG through the hydrolysis of either PC or MGDG and DGDG, which can in turn be converted to PE (Krckova *et al.*, 2015; Cao *et al.*, 2016). The role of *NPC1* in response to heat stress is known (Krckova *et al.*, 2015). Here, we propose a role for *NPC1* in PE accumulation and chilling tolerance in panicoid grasses.

Overall, we show that despite the conservation of many transcriptional and metabolic responses to chilling stress across species, the unique combination of species employed in our study allowed us to identify a smaller set of genes more likely to be functionally linked to variation in chilling tolerance than merely due to genetic relatedness. This study provides a framework to probe potential genes whose function in changes to lipid content and composition may not be previously known. For the first time, we also report diel rhythmicity in lipid abundance, saturation, and expression of lipid-related genes in these panicoid grasses during chilling stress.

## Supplementary data

The following supplementary data are available at *JXB* online.

Fig. S1. Minor lipid responses to chilling include effects related to genetic distance and chilling tolerance.

Fig. S2. Circos plot showing differential expression of sorghum–foxtail millet orthologous genes.

Fig. S3. Venn diagram showing overlap of rhythmic genes under control and chilling stress in sorghum and foxtail millet.

Fig. S4. Total lipid unsaturation in the initial period of chilling stress in foxtail millet, sorghum, and Urochloa.

Table S1. Averages and standard errors of each lipid abundance measurements across different time points in foxtail millet, Urochloa, and sorghum.

Table S2. LimoRhyde test of rhythmicity of measured lipids in three grasses.

Table S3. CircaCompare test of rhythmicity for DGDG, PC, and MGDG in three grasses.

Table S4. Averages and standard errors of each lipid unsaturation across different time points in foxtail millet, Urochloa, and sorghum.

Table S5. FPKM values of 9778 syntenic gene pairs in sorghum and foxtail millet across each time points with triplicates in chilling stress and control.

Table S6. Differentially regulated orthologs (DROs) between sorghum and foxtail millet using a linear mixed model.

Table S7. Sorghum and foxtail millet genes classified into 16 clusters based on the ratios of expression values between control and treatments.

Table S8. Differentially regulated orthologs in sorghum and foxtail millet using conventional clustering method.

Table S9. High confidence DROs identified from the overlap of DROs from the conventional clustering method and the linear mixed model.

Table S10. Gene Ontology (GO) analysis of 1708 high-confidence differentially regulated orthologs.

Table S11. Gene expression profiles of sorghum–foxtail millet orthologous genes corresponding to Arabidopsis lipid-related genes.

Table S12. Log_2_ fold change of differentially regulated orthologs in chilling-treated sorghum and foxtail millet derived from the list of 356 lipid gene pairs in Supplementary Table S9.

Table S13. Pearson correlations of foxtail millet and sorghum genes with changes in buildup of each lipid species during chilling stress response.

Table S14. Pearson correlations of foxtail millet and sorghum genes with changes in breakdown of each lipid species during chilling stress response.

Table S15. Averages and standard errors of lipid accumulation and unsaturation in Arabidopsis.

Table S16. CircaCompare test of rhythmicity for DGDG, PC, and MGDG in Arabidopsis.

Table S17. Averages and standard errors of each lipid accumulation and unsaturation across different time points in control and chilling-treated Arabidopsis.

erae247_suppl_Supplementary_Figures_S1-S4

erae247_suppl_Supplementary_Tables_S1-S17

## Data Availability

Sequencing data are available in the NCBI under accession number SRA: SRP090583 and BioProject: PRJNA344653.

## Abbreviations:

DGDG: digalactosyldiacylglycerol
DRO: differentially regulated ortholog
FDR: false discovery rate
FPKM: fragments per kilobase of transcript per million mapped reads
LMM: linear mixed model
MGDG: monogalactosyldiacylglycerol
PC: phosphatidylcholine
PG: phosphatidylglycerol
TAG: triacylglyceride
